# Breast cancer associated with neurofibromatosis type 1: a case series and review of the literature

**DOI:** 10.1186/s13256-015-0533-8

**Published:** 2015-03-19

**Authors:** Jihane Khalil, Mohamed Afif, Hanan Elkacemi, Meryem Benoulaid, Tayeb Kebdani, Noureddine Benjaafar

**Affiliations:** Radiation Therapy Department, National Cancer Institute, Rabat, Morocco

**Keywords:** Neurofibromatosis, Breast cancer, Uncommon association

## Abstract

**Introduction:**

Neurofibromatosis type 1, also known as Von Recklinghausen’s disease, is a rare neuroectodermal disease that mainly affects the skin and the nervous system. Patients with neurofibromatosis type 1 have a higher risk of developing various types of cancers, especially tumors derived from the embryogenic neural crest. However, its association with breast cancer has seldom been reported.

**Case presentation:**

We report the cases of three white Arabic women diagnosed with neurofibromatosis type 1, with a median age of 40-years-old (range: 39 to 43), who sought treatment at our centre for breast cancer.

**Conclusions:**

The association between neurofibromatosis type 1 and breast cancer is uncommon. In our case series we readdress this association through a literature review.

## Introduction

Neurofibromatosis type 1 (NF1), also known as Von Recklinghausen’s disease, is a rare neuroectodermal disease that mainly affects the skin and the nervous system. It is an autosomal dominant disorder that affects 1 in 3000 individuals [1] and is characterized by cafe-au-lait spots and multiple neurofibromas. NF1 has been reported to be associated with various types of cancers, especially tumors derived from the embryogenic neural crest, including pheochromocytoma, leukemia, glioma, rhabdomyosarcoma, astrocytoma and neurofibrosarcoma [2,3]. Breast cancer is rarely reported in association with NF1. We report the cases of three white Arabic women presenting with breast cancer and a previous diagnosis of NF1, and review the available data in the literature.

## Case presentation

### Case one

**Figure 1:**
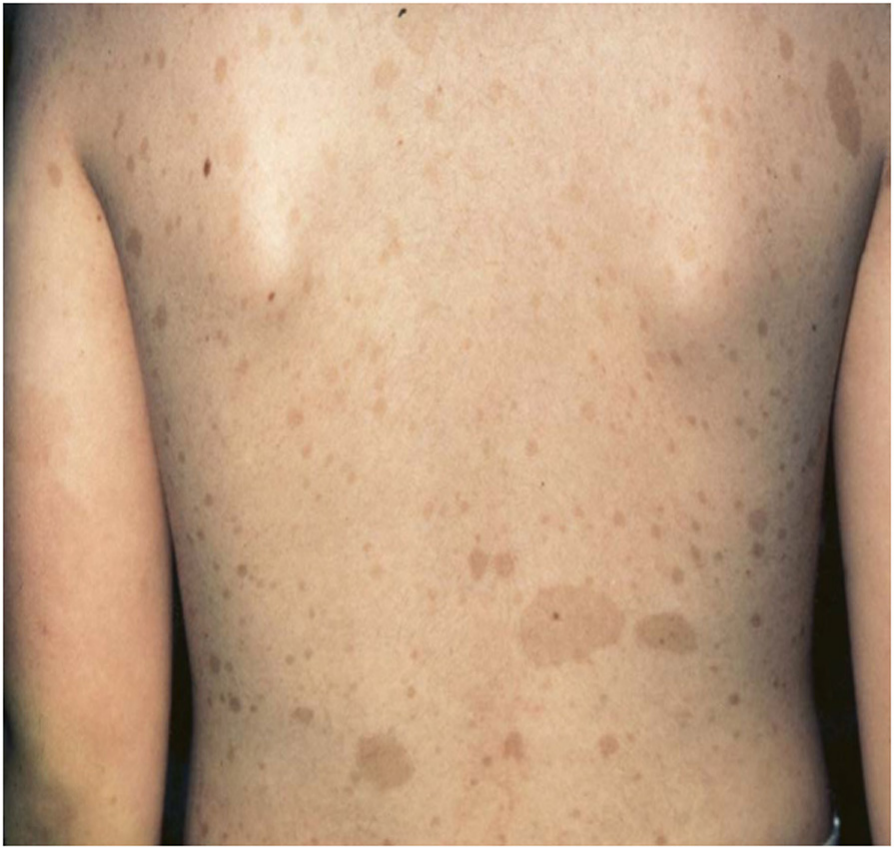
**Case one: photograph of café-au-lait spots.**

A 39-year-old white Arabic woman was referred to our center for adjuvant radiotherapy of a ductal carcinoma in her right breast. She was diagnosed with NF1 by her neurologist when she was eight years old; she was noted to have several cafe-au-lait spots (Figure 1).

She had presented with a large lump in the superior outer quadrant of her right breast one year before her admission to our department. On palpation, this lump was noted to be hard and irregular. A mammography was then performed and suggested the malignancy of the lesion, as it was staged as stage 4 according to the Breast Imaging Reporting and Data System (BI-RADS) established by the American College of Radiology (ACR). Her biopsy was positive for invasive ductal carcinoma and her additional work-up was negative for distant metastasis via bone scan, chest and abdominal computed tomography (CT).

A mastectomy, along with a right axillary lymph node dissection, were performed and the tumor was classified as stage PT3N2M0(IIIA) according to the TNM Staging System for Breast Cancer adopted by the American Joint Committee on Cancer (AJCC), (luminal B, estrogen receptor [ER] positive, progesterone receptor [PR] positive and human epidermal growth factor [HER] 2 positive). She received adjuvant chemotherapy (three courses of FEC (cyclophosphamide, epirubicin and 5-fluorouracil) followed by three courses of docetaxel with trastuzumab (Herceptin®) three weeks after her surgery. Radiotherapy was delivered to her chest wall and regional nodes to a total dose of 42Gy after completion of chemotherapy. She was started on adjuvant tamoxifen (20mg, orally) immediately after radiation and was asked to continue this treatment for five years. After 28 months, she remains well with no signs of recurrence of her breast cancer.

### Case two

**Figure 2:**
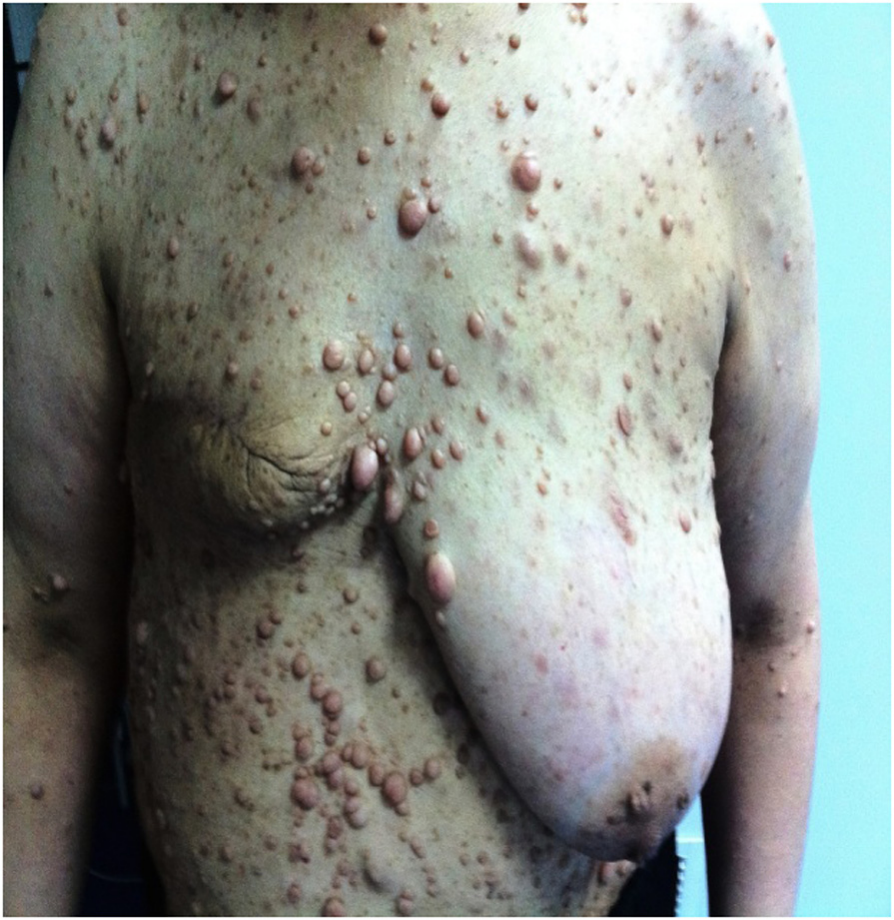
**Case two: photograph of neurofibromas in the trunk and the limbs after mastectomy.**

A white Arabic 40-year-old woman presented to our center with cancer of her left breast. She was diagnosed as having NF1 at seven years of age; she had the classical form of neurofibromatosis, with multiple nerofibromas over her limbs and trunk (Figure 2).

With no family history of breast cancer, she was diagnosed at 40-years-old with cancer of her left breast. Her mammography showed an irregular 8cm lump in the retro-areolar area, classified as stage ACR5. A biopsy of the lesion confirmed its malignancy, and the histological type was invasive ductal carcinoma.

After the weekly team board meeting, a radical mastectomy with homolateral axillary nodes dissection was recommended. Her tumor was classified as stage PT3N3M0 (IIIC), according to the pathologic findings, and it was a triple negative. An adjuvant chemotherapy and radiotherapy was delivered. She received three cycles of anthracycline followed by three cycles of docetaxel. Radiation therapy was delivered to her left chest wall along with regional lymph nodes to a total dose of 42Gy. At 30 months after her mastectomy she exhibits no evidence of recurrence.

### Case three

We present the case of a 43-year-old white Arabic woman whose diagnosis of NF1 was made soon after birth, when she was noted to have numerous cafe-au-lait spots and progressively developed axillary lentigines and neurofibromatous lesions on her trunk and limbs. At 43 years old she noted a mobile lump in her upper internal left breast quadrant upon self-examination. A biopsy was taken and tested positive for invasive ductal carcinoma. She received the same treatment as patients in cases one and two as her tumor was also classified as stage PT3N2M0 (IIIA). Adjuvant systemic hormonal therapy was prescribed for five years as she was ER and PR positive. At two years after her mastectomy she remains free of any local recurrence or distal metastasis.

## Discussion

NF1 is a complex neuroectodermal disorder characterized by its autosomal dominant inheritance, high penetrance and a wide variability in expression. The *NF1* gene is located in the peri-centromeric region of the long arm of chromosome 17 (which also houses the *BRCA1* gene). It regulates the conversion of the active Ras-GTP to inactive Ras-GDP. Ras is known as an essential component of signal transduction pathways that regulate growth, proliferation, differentiation, and apoptosis. The conversion from the GTP- to the GDP-bound form is mediated by the intrinsic GTPase activity of Ras. The impairment of this hydrolytic reaction is associated with an increased risk of cancer [3]. Hence, it has a potential role as a tumor suppressor gene [4]. The association between NF1 and malignant tumors has been widely described; the most common reported associations are with gliomas, malignant peripheral nerve sheath tumors (MPNST), leukemia and rhabdomyosarcoma [2,3]. Concerning the association between NF1 and breast cancer, only a few cases have been reported [5,6]. Interestingly, about 28% of sporadic breast cancers are missing at least one copy of the *NF1* gene, either due to deletion or mutation [7].

Clinically, NF1 is recognized mostly by multiple neurofibromas, café-au-lait spots and Lisch nodules [2,3]. The National Institutes of Health in the United States defined seven eligible criteria by which to diagnose NF1; the diagnosis of NF1 is established whenever two signs are associated in the same individual (Table 1) [8].

**Table 1:** **Diagnostic criteria for neurofibromatosis type 1 according to the National Institutes of Health** [8]

**Diagnostic criteria for neurofibromatosis type 1**	
1.	Six or more café-au-lait macules over 5mm in diameter in prepubertal individuals and over 15mm in diameter in postpubertal individuals.
2.	Two or more neurofibromas of any type or one plexiform neurofibroma.
3.	Freckling in the axillary or inguinal region.
4.	Optic glioma.
5.	Two or more Lisch nodules (iris hamartomas).
6.	A distinctive osseous lesion such as sphenoid dysplasia or thinning of the long bone cortex with or without pseudarthrosis.
7.	A first-degree relative (parent, sibling or offspring) with neurofibromatosis type 1 as diagnosed by the above criteria.

The first cases describing the association of NF1 with breast cancer were reported in the 1970s by Brasfield and Das Gupta. They described their experience with five patients, including one who had bilateral breast cancer [9].

Since then many case reports have been published. Murayama *et al.* [10] reported 37 cases of breast cancer associated with NF1; most of the cases were diagnosed at an advanced stage and had invasive ductal carcinoma. The authors explained the advanced stage at the diagnosis by the presence of cutaneous fibromatas in the trunk in most of the patients which could have delayed the early diagnosis. In our cases, breast cancer was diagnosed at an advanced stage in all of our patients (stage IIIA in two cases and IIIC in one case).

In an earlier report by Nakamura *et al.* [5], the authors noted that breast cancer affected young women (<35 years old), in 18.5% of the cases which is relatively high when compared to the findings of other series of breast cancer not associated with NF1, which reported a percentage of 6.7%. In our patients, breast cancer was also diagnosed at a young age (median age of 40 years old).

In a study of Sharif *et al.* [11], the main objective was to evaluate the risk of developing breast cancer among patients with NF1. A cohort of 304 women aged 20 years and above who were diagnosed with NF1 was studied over a period of 30 years. The authors reached the conclusion that women with NF1 had five times more chances of developing breast cancer when compared to the general population. Concluding that, although reported cases are rare, the association between breast cancer and NF1 is common and patients with NF1 have a moderately elevated risk of developing breast cancer [11]. Despite these findings, patients with NF1 are still not stratified as high-risk patients, and current guidelines do not give specific considerations regarding any screening program for this category of patients [12,13].

Treatment of this particular population with breast cancer is conflicting. Even if a conservative approach is considered to be convenient, there have been reports of an increased risk of developing fibrosarcoma secondary to radiation therapy when indicated [14,15]. Additionally, silencing the *NF1* gene has been shown to confer tamoxifen resistance in human breast cancer (MCF7) cell lines [16]. Hence, there is a growing hypothesis that tamoxifen might be less effective in cancers involving NF1 mutations; however, clinical evidence is missing.

As for the general population with breast cancer, women with NF1 can also be candidates for breast reconstruction following surgery. In the literature we found a case report by Yamamoto [17] in which a 33-year-old woman had undergone reconstructive surgery using the abdominal rectus muscle.

## Conclusions

Even if the association between breast cancer and NF1 is rarely reported, the few studies found in the literature suggest that women with NF1 are at a higher risk of developing breast cancer when compared to the general population. However, there are no specific considerations for screening in this particular population. Moreover, cancer management in this population is not well defined; especially when some available data suggests that the risks of fibrosarcomas are increased by radiation when a conservative approach is chosen for this population.

## Consent

Written informed consent was obtained from the patients for publication of this case report and any accompanying images. A copy of the written consent is available for review by the Editor-in-Chief of this journal.

## Abbreviations

NF1: Neurofibromatosis
BIRADS: Breast imaging reporting and data system
AJCC: American joint committee on cancer
E.R.: Estrogen receptor
P.R.: Progesterone receptor
HER: Human epidermal growth factor
MPNST: Malignant peripheral nerve sheath tumors
